# Jewelry rock discrimination as interpretable data using laser-induced breakdown spectroscopy and a convolutional LSTM deep learning algorithm

**DOI:** 10.1038/s41598-024-55502-x

**Published:** 2024-03-02

**Authors:** Pouriya Khalilian, Fatemeh Rezaei, Nazli Darkhal, Parvin Karimi, Ali Safi, Vincenzo Palleschi, Noureddine Melikechi, Seyed Hassan Tavassoli

**Affiliations:** 1 Department of Physics, K. N. Toosi University of Technology, Tehran, 15875-4416 Iran; 2 Research Institute of Conservation and Restoration, Research Institute of Cultural Heritage and Tourism, Tehran, Iran; 3 Department of Physics, South Tehran Branch, Islamic Azad University, Tehran, Iran; 4 Physics and Applied Physics, Kennedy College of Sciences, University of Massachusetts Lowell, Lowell, USA; 5 Institute of Chemistry of Organometallic Compounds Research Area of CNR, 56124 Pisa, Italy; 6 Laser and Plasma Research Institute, Shahid Beheshti University, Tehran, Iran

**Keywords:** Deep learning, LIBS spectroscopy, Jewelry rock, Convolutional LSTM, Chemometrics, Applied optics, Lasers, LEDs and light sources, Optical physics

## Abstract

In this study, the deep learning algorithm of Convolutional Neural Network long short-term memory (CNN–LSTM) is used to classify various jewelry rocks such as agate, turquoise, calcites, and azure from various historical periods and styles related to Shahr-e Sokhteh. Here, the CNN–LSTM architecture includes utilizing CNN layers for the extraction of features from input data mixed with LSTMs for supporting sequence forecasting. It should be mentioned that interpretable deep learning-assisted laser induced breakdown spectroscopy helped achieve excellent performance. For the first time, this paper interprets the Convolutional LSTM effectiveness layer by layer in self-adaptively obtaining LIBS features and the quantitative data of major chemical elements in jewelry rocks. Moreover, Lasso method is applied on data as a factor for investigation of interoperability. The results demonstrated that LIBS can be essentially combined with a deep learning algorithm for the classification of different jewelry songs. The proposed methodology yielded high accuracy, confirming the effectiveness and suitability of the approach in the discrimination process.

## Introduction

Laser induced breakdown spectroscopy (LIBS) is an analytical technique that provides multi-elemental qualitative and quantitative information with relatively high sensitivity and spatial resolution^1–4^. In LIBS technique, a pulsed laser, typically a Q-switched Nd:YAG laser, is used to vaporize a small amount of material, creating a hot plasma that emits characteristic radiation. This plasma radiation reveals the elemental composition of the sample by its unique spectral signature^5^. The increasing interest in LIBS has prompted researchers to explore various methodologies aimed at enhancing the analytical capabilities of this technique^6–9^. LIBS has been used in many fields. These range from space, industry, pollution, medical, to cultural studies^10–15^. In the field of archaeological science and geology, in particular, several of LIBS features are of great significance. Its ease of use, speed of analysis, and the lack of sample preparation requirements make it possible to perform in-situ analysis. Applications of LIBS in various areas of archeology have been recently reviewed^16,17^.

Recent advances in artificial intelligence offer further potential to enhance the capabilities of LIBS in various applications. Deep learning methodology, which illustrates a broad category of machine learning algorithms based mainly on Artificial Neural Networks, has become the most studied in the artificial intelligence field^18^. Convolutional Neural Network (CNN) is a deep learning technique used in data fitting and feature learning, and most notably in spectral analysis^19^. Several research groups have applied convolutional deep learning algorithms to LIBS data acquired on various types of samples. In the cultural sphere, Pierdicca et al.^20^ applied a deep learning framework for segmentation of Point Cloud. They used the dynamic graph of the Convolutional Neural Network algorithm by considering features like color and normal. Llama et al.^21^ classified the images obtained during the measurement of an architectural asset through Convolutional Neural Networks. They showed that this method can be applied employed in the digital documentation of architectural heritage. In addition, Chen et al.^22^ combined the deep Convolutional Neural Network with the fast multi-element compositional imaging capability of LIBS and obtained a 100% classification accuracy of the rock lithology of shale, gneiss, and granite.

Shahr-e Sokhteh (The Burnt City) is one of the symbols of the great civilization of Sistan plain in Iran. It is the name of a hill or a series of interconnected wide hills that are located about 56 km from the Zabol-Zahedan road and southeast of Zabol city. The location of Iran between two advanced centers of civilization in the third millennium BC, namely Mesopotamia, the Elamite, and Sumerian government in the west, Hindu Harappa, and the Indus Valley in the east, demanded that the people of this region be connected to other cultures and civilizations, particularly Asian cultures in East and West Asia, but most of these connections were provided through trade highways in the form of trade in goods^23^.

The results of studies and experiments performed on cultural materials obtained from this city revealed four cultural-settlement periods for this city^24,25^. The beginning of the first historical period is attributed to 3200 BC, which is the oldest known settlement date in the Shahr-e-Sokhteh and Sistan plains. These hills were first identified by Stein in 1916 and excavated by Italian archaeologists from the Iziao Institute during 1968–1978^24,25^. Excavations in the eastern residential area were carried out by Maurizio Tuzi, who was also in charge of the excavations^25^. Excavations in the central part were carried out by Massimo Vidal and Sandro Salvatori^24^. The second period of excavations in this area has been excavated by a group of Iranian archaeologists led by Dr. Seyed Mansour Seyed Sajjadi since 1997^26^.

It should be noted that Shahr-e Sokhteh is one of the most significant and key Bronze Age sites in the archeology of southeastern Iran^27–30^. The excavations of these sites performed over the years, revealed the presence of a large number of semi-precious stones and jewels. It should be mentioned that some of the excavated healthy beads were in the form of necklaces, bracelets, and bindings, and some others were in the form of semi-finished beads, raw stones, as well as unworked blocks^31^. These jewels are azure, agate (blue, red, yellow, smoky, solid, and colorless agate), chlorite, turquoise (blue and green), limestone, flint, jasper, marble (calcite and aragonite), quartz, green tuff, and chert stone.

In this research, a LIBS experiment is performed for the discrimination of jewelry rock, including agates, turquoises, calcites, and azures, that belong to the historical place of Shahr-e Sukhteh in Iran. Furthermore, LSTM method is combined with Convolutional Neural Network to discriminate various gemstones. Generally, LSTM method manage.

the memory information of the data, comprising time series problems. Here, LSTM method as a deformation structure of a recurrent neural network (RNN) inserted memory cells into the hidden layer by several programmable gates of forget gate, input gate, and output gate to transmit information among multiple hidden layer cells.

## Materials and methods

**Figure 1:**
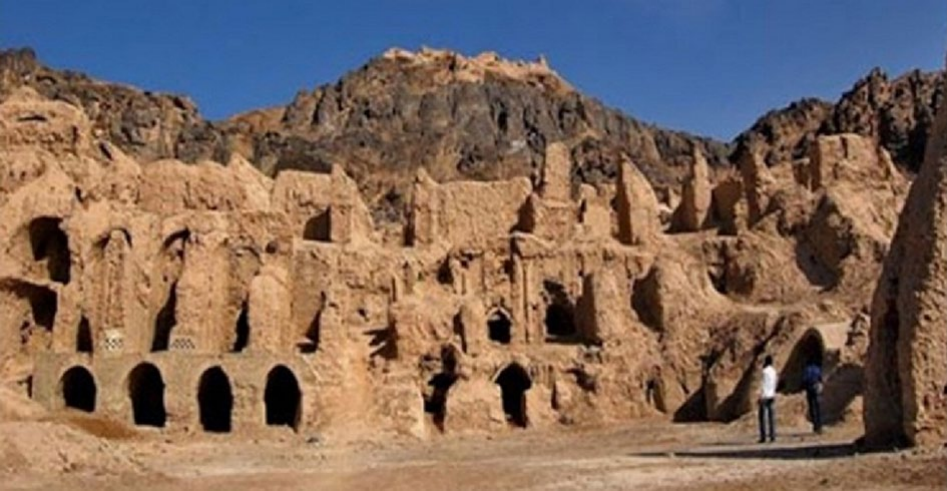
A picture of Shahr-e Sokhteh in Iran. Reprinted from Persian touring site, with permission from^32^.

Figure 1 depicts a clear image of Shahr-e Sokhteh, including various hills. The people of Shahr-e Sokhteh, like other communities, have paid attention to the beauty of the exterior and interior of their bodies. One of the tasks of the bead makers of the Shahr-e Sokhteh was to prepare and make all kinds of beads from materials such as wood, bone, mud, pottery, stone, and metal for use in funeral ceremonies. The same artisans obtained other materials, such as precious and semi-precious stones, by burning them in different parts of the city, especially in the city cemetery. The presence of stones and semi-finished beads, as well as beadwork tools on the surface, and the remains of industrial and masonry workshops, as well as those of other workshops, including pottery, all contribute to this impression. Manufacture and metalwork in the Shahr-e Sokhteh’s industrial area have demonstrated the construction and payment of objects in that location.

**Figure 2:**
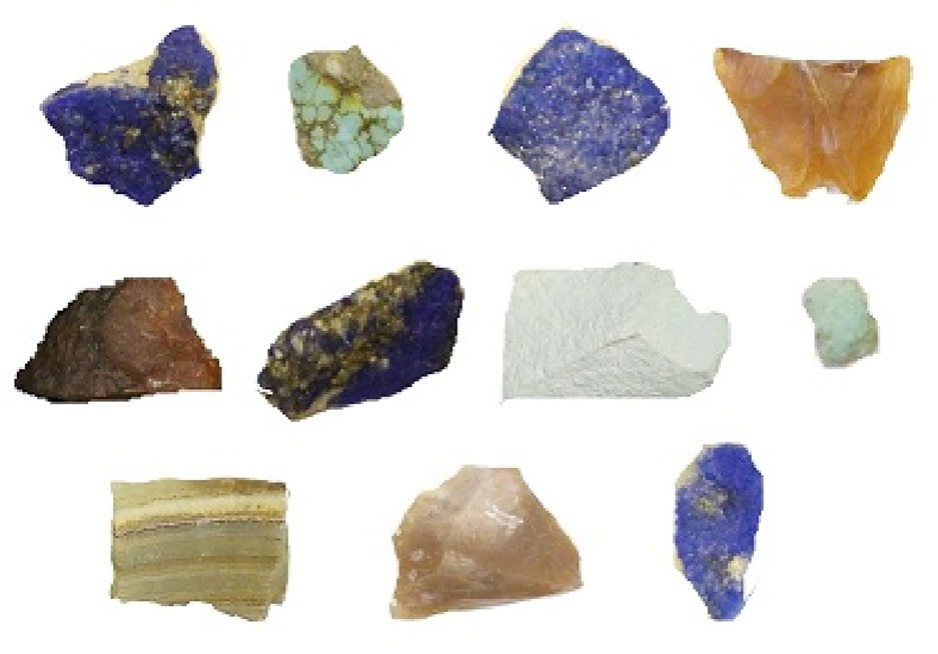
Different jewelry songs related to Shahr-e Sokhteh for deep learning analysis.

Semi-precious stones in Shahr-e Sokhteh were made from a variety of sources, and in various workshops they were constructed into different objects. Apart from the azure and turquoise stones, almost all the raw materials used in the city were supplied from the heights besieged by Sistan. For instance, the agate was obtained from the riverbed, and the rest of these raw materials were supplied from the Chagai area, located at the southern end of the old delta. In this paper, different gemstones from Shahr-e Sokhteh, including 12 agates, 3 turquoises, 2 calcites, and 2 azures, were collected for analysis, as shown in Fig. 2.

### Experimental set-up (LIBS spectroscopy)

**Figure 3:**
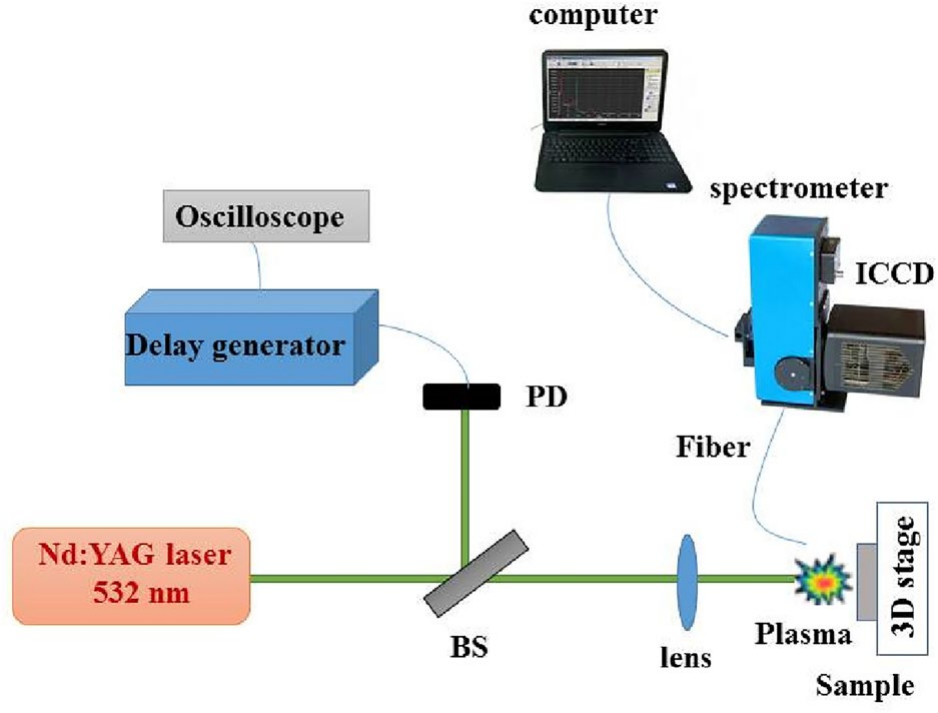
A schematic of the experimental set-up of the LIBS method for the analysis of jewelry rocks.

A schematic diagram of a LIBS experimental set-up for spectrum acquisition is presented in Fig. 3. A Q-switched Nd:YAG laser pulse (Continuum, Surelite III) at 532 nm wavelength, with a repetition rate of 5 Hz, and pulse duration of 10 ns is irradiated on different gemstones^33,34^. The samples are 12 agates, 3 turquoises, 2 calcites, and 2 azures jewelry rocks placed on a motorized micrometric XYZ stage. Generally, a gemstone or jewelry rock is a piece of mineral crystal that can be cut or polished for use in jewelry applications or other adornments**.** Here, a lens with a focal length of 80 mm concentrates the irradiation on the targets. The plasma emission is collected by employing a quartz lens and is directed into an optical fiber coupled into an Echelle spectrometer (Kestrel, SE200). The spectral range of this system is 190 to 950 nm. The temporal analysis of the recorded spectra is investigated by changing the gate and delay time of the ICCD camera (Andor, iStar DH734). During the LIBS experiments, the delay time and gate width are adjusted for 1 μs by using a digital delay generator (model Stanford DSG 535), and the laser energy is considered to be 80 mJ after optimization to enhance the signal-to-noise ratio (SNR)^35–38^.

It should be noted that each spectrum is an accumulation of 10 laser pulses. In this paper, normalization is performed in the pre-processing of LIBS spectra data due to the fluctuation of matrix characteristics and laser energy. It should be mentioned that there is complexity in the matrix composition of rocks, and the emitted spectral frequency and intensity are various for different species of LIBS plasma.

The various peaks of the LIBS spectral intensities related to the different elements are used as features for classification. Moreover, the type of stone corresponding to each entry is selected as a label. To change the learning data as into interpretable information and, on the other hand, contain the necessary dynamics for modeling with the LSTM method, a trick of combining the spectra of each element for the tensor input is applied. As a result of this combination, the number of data has reached 43,315 combined spectra. Consequently, the spectra are used for the determination of the type of rock through the LIBS spectrum, which makes the data interpretable for modeling.

### CNN–LSTM algorithm

Due to the success of deep learning in chemometrics analysis, many researchers have been attracted to deep learning. Convolutional neural network (CNN) and long short-term memory (LSTM) networks are widely used techniques in deep learning algorithms^39^. The main goal of applying these methods to time-series data is that the LSTM model has the capability of capturing the sequence pattern data, while the CNN method is beneficial in extracting precious features that may filter the noise related to the input data. The main difference between LSTM networks and CNNs is that LSTM networks work on temporal correlations and use only the attributes provided in the training set, whereas CNNs are utilized for obtaining patterns of local trend, and as well as similar patterns emerging in various regions of time-series data that are not typically adjusted for long temporal dependencies. Consequently, the combination of the advantages of both deep learning models will enrich the forecasting accuracy. In this study, the deep learning methodology of CNN–LSTM assisted to the analysis of the complex phenomena such as the optical emission of the laser-induced plasmas. Here, CNN–LSTM is developed for application to a discrimination problem related to the LIBS technique.

### CNN network

**Figure 4:**
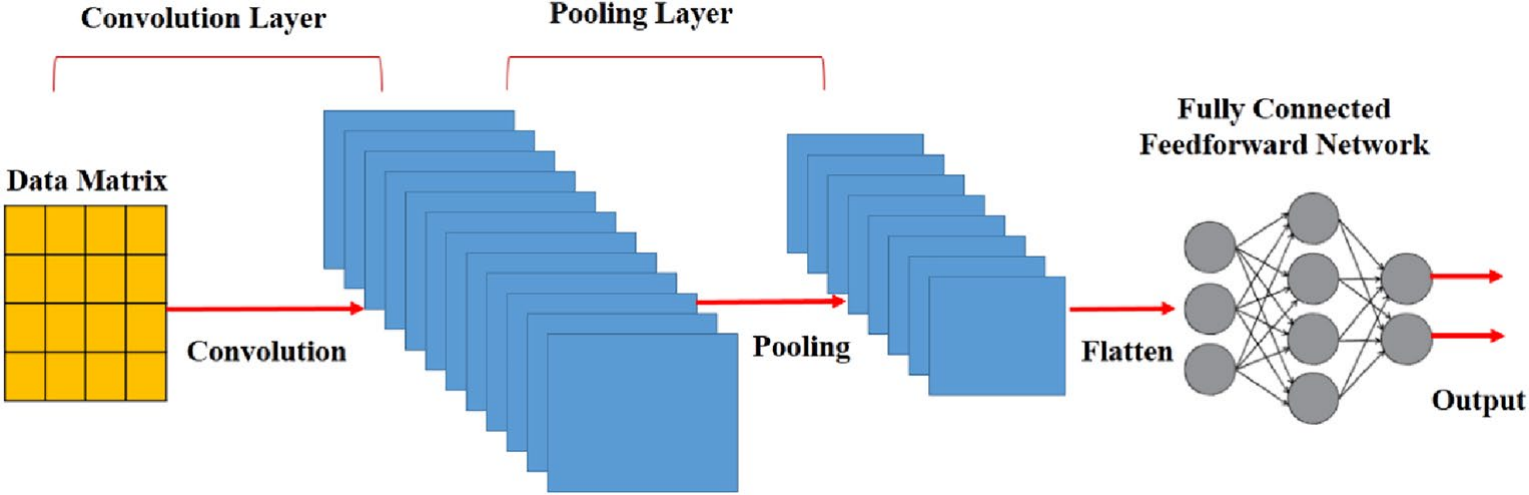
General structure of a CNN neural network^41^.

Convolutional Neural Network is a feedforward neural network with a deep configuration, that is frequently applied to image processing problems^39,40^. The typical structure of CNN network is presented in Fig. 4. This figure shows that CNN contains four different layers of data matrix input, pooling, convolution, and a fully connected layer^41^.

The core structure of a typical CNN network is the convolution operation. It should be noted that its difference from the fully connected structure is that the convolution operation comprises the entire advantage of the information related to the neighborhood regions of the data matrix. Sparse connections and sharing weights significantly reduce the size of the parameter matrix^42^. In addition, the pooling layer generates its unique feature map during the acquisition of the average or maximum data, which obtains feature compression and prevents overfitting up to a certain value. It should be noted that multi-layer convolution and pooling operations can be built into CNN networks. A higher level of abstraction of features can be obtained in a deeper layer of the neural network's structure. A fully connected layer combines the extracted abstract features, and the regression and classification problems are solved using a softmax or sigmoid activation function^43^. In this paper, the one-dimensional CNN network is applied to extract the spatial feature of the trajectory data.

### LSTM model

**Figure 5:**
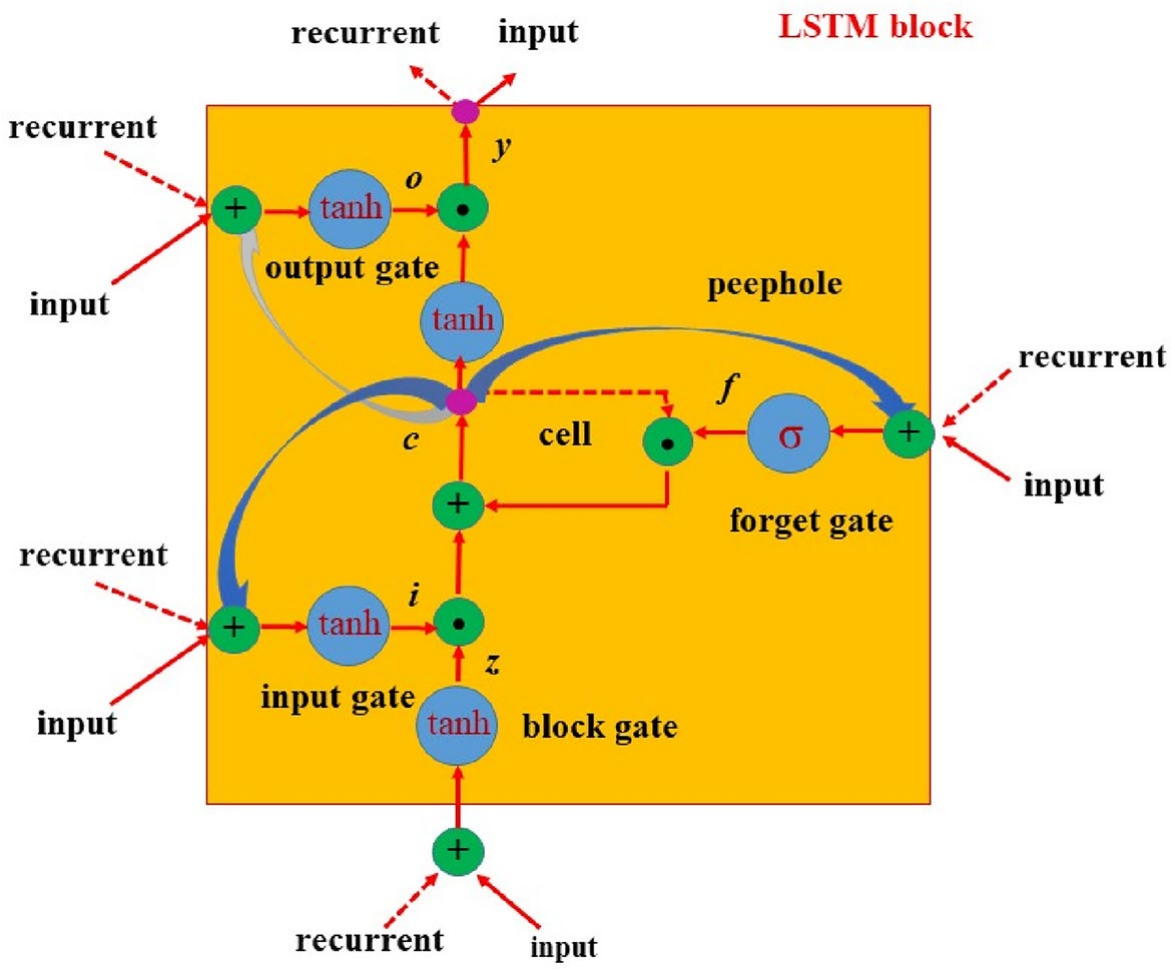
Long short-term memory method^46^.

Long short-term memory (LSTM)^44,45^ is a deformation structure of Recurrent Neural Network (RNN) that adds memory cells into the hidden layer to manage the memory information of the data, including time series problems. Information is transmitted among various hidden layer cell by using different controllable gates (forget gate, input gate, and output gate), as shown in Fig. 5^46^. The memory cell's state is controlled by two gates: forget and input. The forget gate determines how much "memory" of the previous cell can be stored. In addition, the input gate indicates how much input from the present moment can be saved to the cell state, and controls the contribution of fusion of the ‘‘historical’’ information and ‘‘recent’’ stimulus. The output gate of LSTM controls how much information is output for cell status. The essential improvement of LSTM in comparison to traditional RNN is the presentation of different gating mechanisms that control the memory and forget previous and current information. Furthermore, LSTM comprises the long-term memory function compared to a standard RNN, and the problem of its gradient disappearance is also prevented.

The input and output of the LSTM network structure are expressed in Eqs. (1)–(8)^35^:

1 $${\text{Input}}\;{\text{gate}}:F(t) = \sigma \left( {W_{f} \cdot \left[ {H_{t - 1} ,X_{t} } \right] + b_{f} } \right) \oplus$$

2 $${\text{Forget}}\;{\text{gate}}:I(t) = \sigma \left( {W_{i} \cdot \left[ {H_{t - 1} ,X_{t} } \right] + b_{i} } \right)$$

3 $${\text{Memory}}\;{\text{cell}}:\tilde{C}(t) = \tanh \left( {W_{c} \cdot \left[ {H_{t - 1} ,X_{t} } \right] + b_{c} } \right)$$

4 $$C(t) = f_{t} * C_{t - 1} + I_{t} * \tilde{C}_{t}$$

5 $${\text{Output}}\;{\text{gate}}:O(t) = \sigma \left( {W_{0} \cdot \left[ {H_{t - 1} ,X_{t} } \right] + b_{0} } \right)$$

6 $$H(t) = O_{t} * \tanh (C_{t} )$$

7 $$sigmoid(x) = \frac{1}{{1 + e^{ - x} }}$$

8 $$\tanh \,(x) = \frac{{e^{x} - e^{ - x} }}{{e^{x} + e^{ - x} }}$$

here *W*_*f*_, *W*_*i*_, *W*_*c*_ and *W*_*0*_ are input weights. *b*_*f*_, *b*_*i*_, *b*_*c*_ and *b*_*0*_ are bias weights. Moreover, *t* and *t*−1 are the present and previous time states, respectively. *X*_*t*_ indicates input, *H*_*t*_ shows output, and *C*_*t*_ represents the cell status at time *t*. *σ* represents a sigmoid activation function with output values between 0 and 1, where 0 indicates “let nothing pass”, and 1 means “allow everything pass”. In Eq. (8), the hyperbolic tangent function is inserted to overcome the gradient disappearance’s difficulties. Furthermore, *f*_*t*_ presents the forget gate, and *I*_*t*_ is the input gate. It should be mentioned that in the above equations, at each moment, the duty of the forget gate is to control the extent of memory forgotten at the last moment, and the input gate manages the extent of new memory $\tilde{C}_{t}$ written to the long-term memory. *O*_*t*_ indicates the output gate that controls the influence of long-term memory on short-term memory.

### LASSO method

Least absolute shrinkage and selection operator, known as LASSO regression analysis technique in machine learning and statistics combine variable selection and regularization to enhance the predictability and understandability of the generated statistical models. LASSO calculates a vector of regression coefficients by minimizing the residual sum of squares, while being constrained by the *l*^1^-norm of the coefficient vector. If the sum of the absolute values of the coefficients is less than a constant, LASSO optimizes the residual sum of squares when selecting variables.

More specifically^47^:

9 $$\hat{\beta }^{L} = argmin\left\{ {\mathop \sum \limits_{i = 1}^{n} \left( {y_{i} - \alpha - \mathop \sum \limits_{i = 1}^{n} \beta_{j} x_{ij} } \right)^{2} } \right\}.$$

Assuming that $\sum\nolimits_{j = 1}^{p} {\left| {\hat{\beta }_{j}^{L} } \right|} \le c\left( {Constant} \right)$. Here, α is the constant coefficient, and β_j_ is the coefficient vector.

This issue can be stated as bellows^47^:

10 $$\hat{\beta }^{L} = argmin\left\{ {\mathop \sum \limits_{i = 1}^{n} \left( {y_{i} - \alpha - \mathop \sum \limits_{i = 1}^{n} \beta_{j} x_{ij} } \right)^{2} + \lambda \mathop \sum \limits_{j} \left| {\beta_{j} } \right|} \right\}$$

here, λ > 0 is chosen so that $\sum\nolimits_{j = 1}^{p} {\left| {\hat{\beta }_{j}^{L} } \right|} = c\left( {Constant} \right)$, and each λ is turning factor which equal to a various Lasso parameter *c*^48^. When the LASSO value is small enough, some regression coefficients reach zero. Because of this, the LASSO algorithm only selects a subset of the regression coefficients for each LASSO algorithm. The LASSO parameter *c* > 0 determines how much shrinkage is applied to the estimation.

### Data features and designing an interpretable dataset

In this paper, 43 spectra of agate, 20 spectra of calcite, 59 spectra of turquoise, and 46 spectra of lapis lazuli are employed for statistical analysis. Each spectrum, which is an accumulation of 10 laser pulses, is taken from each gemstone, and the intensity peak of each normalized spectrum is considered as a feature. Accordingly, the problem of data normalization is that the peak of each spectrum is the same for different elements, which causes the data to be uninterpretable for modeling. The algorithm, on the other hand, uses a Convolutional LSTM Neural Network for modeling to determine the examined memory. Furthermore, the data related to each gemstone has been combined so that instead of one input spectrum, two input spectra are employed for classification. The advantage of the present study is that it makes the input data of the network interpretable and produces a significant increase in the input data for modeling, which causes better learning of the Recurrent Neural Network model.

It should be mentioned that after combining the data related to each gemstone, the results of number of spectra before combining and number of features after combining will be obtained, which are shown in Table 1. As it is clearly seen in this table, after combining, 7678 interpretable preprocessed data are prepared for modeling. Here, 10 different intensities are obtained from measuring the spectrum and 1 feature is related to the identified element by LIBS method.

**Table 1:** Data characteristics related to different gemstones for statistical modeling in two cases of before and after combining.

Stone	Agate	Calcite	Turquoise	Lapis lazuli
Number of spectra before combining	43	20	59	46
Number of features after combining	1806	380	3422	2070

### Network topology and hyper parameter selection in CNN–LSTM

After designing the dataset in an interpretable manner, the CNN–LSTM architecture is employed as the topology for classification. Table 2 represents the topology of CNN–LSTM for modeling, including the different layers’ features. In this research, the Kernel hyperparameter is equal to 3 for the convolutional layer, 6142 data are considered as training data, and 1536 data are employed as the test data. Furthermore, the Adam optimization function with a learning rate of 0.0005 has been used as an optimization hyperparameter. Moreover, the categorical cross-entropy cost function is employed to calculate loss. In this case, the epoch’s value is 100, the batch size is 128 and the importance of the validation split is 0.25. In addition, the first layer is the convolutional layer with Relu activation function. The second layer is a dynamic layer that utilizes the LSTM architecture with the tanh activation function. It should be noted that these two layers are responsible for feature extraction. The next layers for classification are three perceptron layers. The perceptron ocher layer has four outputs, and the probability of the input data is assigned to each gemstone. Due to the continuous definition of the output as a probability, the interpretability of the output results can be observed.

**Table 2:** Topology of the CNN–LSTM architecture for data classification.

Layers	Neurons	Activation function	Computational parameters
Input layer	(None, 22, 1)	–	0
Conv1D	(None, 20, 128)	Relu	512
LSTM	(None, 256)	tanh	394,240
Dense	(None, 512)	tanh	131,584
Dense	(None, 256)	tanh	131,328
Dense	(None, 4)	SoftMax	1028
Total params: 658,692			
Trainable params: 658,692			
Non-trainable params: 0			

## Results and discussion

**Figure 6:**
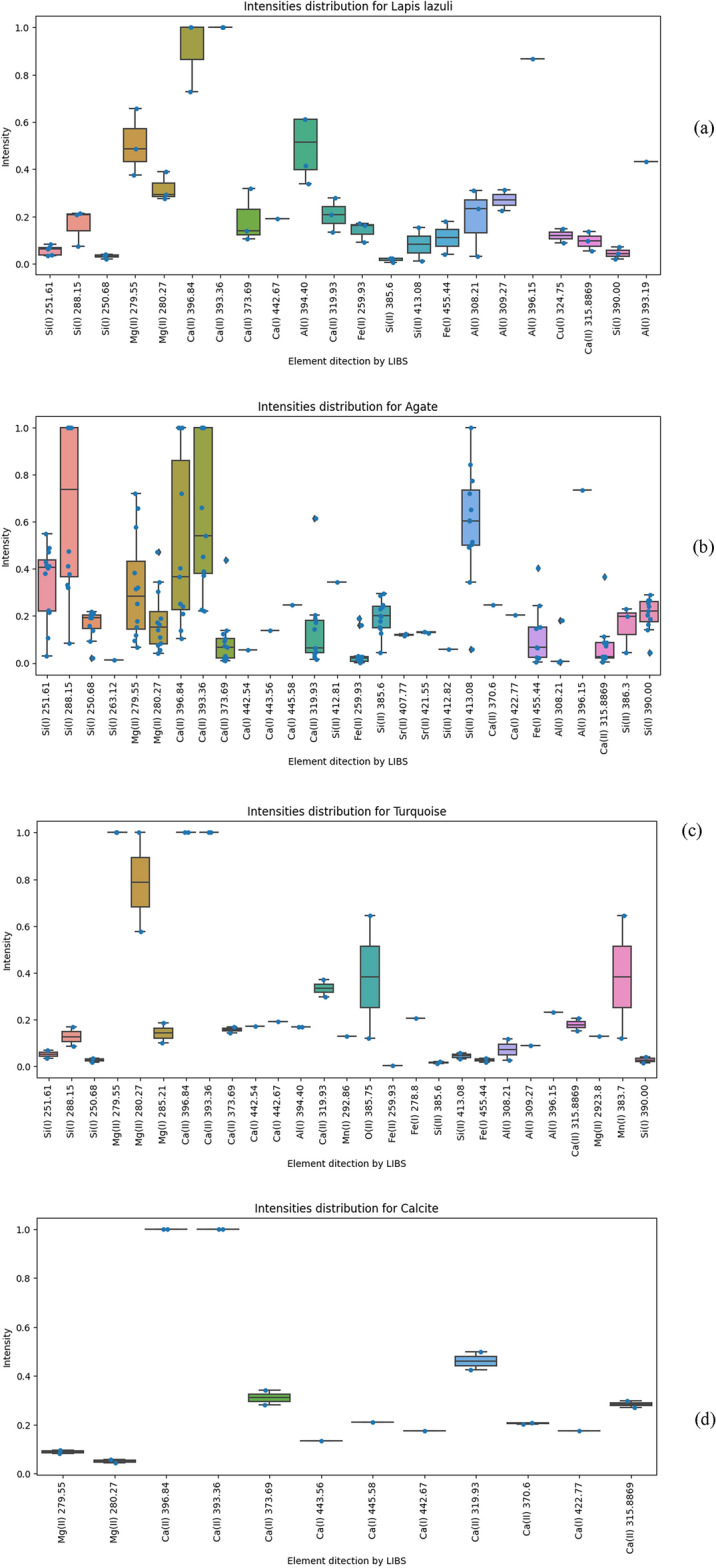
Intensity distribution for different gemstones of (**a**) agate, (**b**) calcite, (**c**) turquoise, and (**d**) lapis lazuli as a function of the constructed elements.

We have analyzed our data by first averaging and normalizing the LIBS spectra of different gemstones is depicted. Different elements in these spectra are identified using the NIST atomic line database. Figure 6 illustrates the scatter plots of the normalized line intensities related to different elements of various jewelry stones, including agate (a), calcite (b), turquoise (c), and lapis lazuli (d). Figure 6 shows that there are common elements among different jewelry stones such as Mg, Ca, Si, Fe, and Al. As it is clearly seen in these figures, the main elements of agate in Shahr-e Sokhteh are Si, and Ca. Furthermore, Ca is the most abundant element in calcite stone, as are Mn and Mg in turquoise stone. In addition, lapis lazuli has some fundamental elements of Al, and Ca. Generally, these gemstones are frequently easily recognizable by people since they have distinctive aesthetic characteristics, but in some cases, due to complex structures and colors, they can’t readily be distinguished. The visual variability of these gemstones may be reflected in the lower prediction results for other stone compositions.

This section summarizes the findings from the experiments carried out utilizing our suggested methodology. The 1D CNN–LSTM network model using the Python Neural Networks library and Keras running on the TensorFlow 2.0 Python development environment were used to train the data. The results of the accuracy and loss calculations related to the training and test data modeling are shown in Table 3. It should be noted that in the classification problems, accuracy is the evaluation criterion. According to this table, the discrimination accuracy of the present model on the training set is 89.8%, but the discrimination accuracy on the test set is 96.4%.

**Table 3:** Result of the accuracy and loss of the test and training data related to the CNN–LSTM network modeling.

Data	Accuracy (%)	Loss
Train	89.8	0.344
Test	96.4	0.092

In this study, by changing the network's hyper-parameters, such as the optimization method, learning rate, and number of epochs, the accuracy is improved. Additionally, by utilizing various methods like data augmentation^49^ and generative adversarial networks^50^ which assist in expanding the amount of training data, the classification accuracy may also be enhanced.

**Figure 7:**
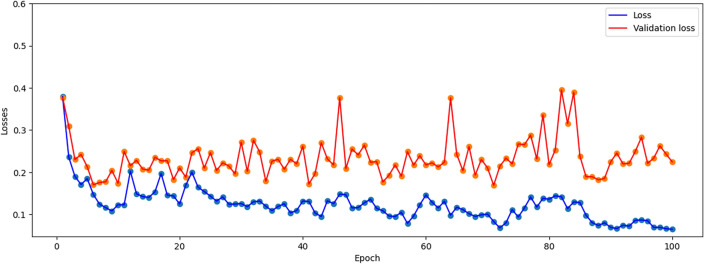
The evolution of losses versus epochs for both the training and test data.

**Figure 8:**
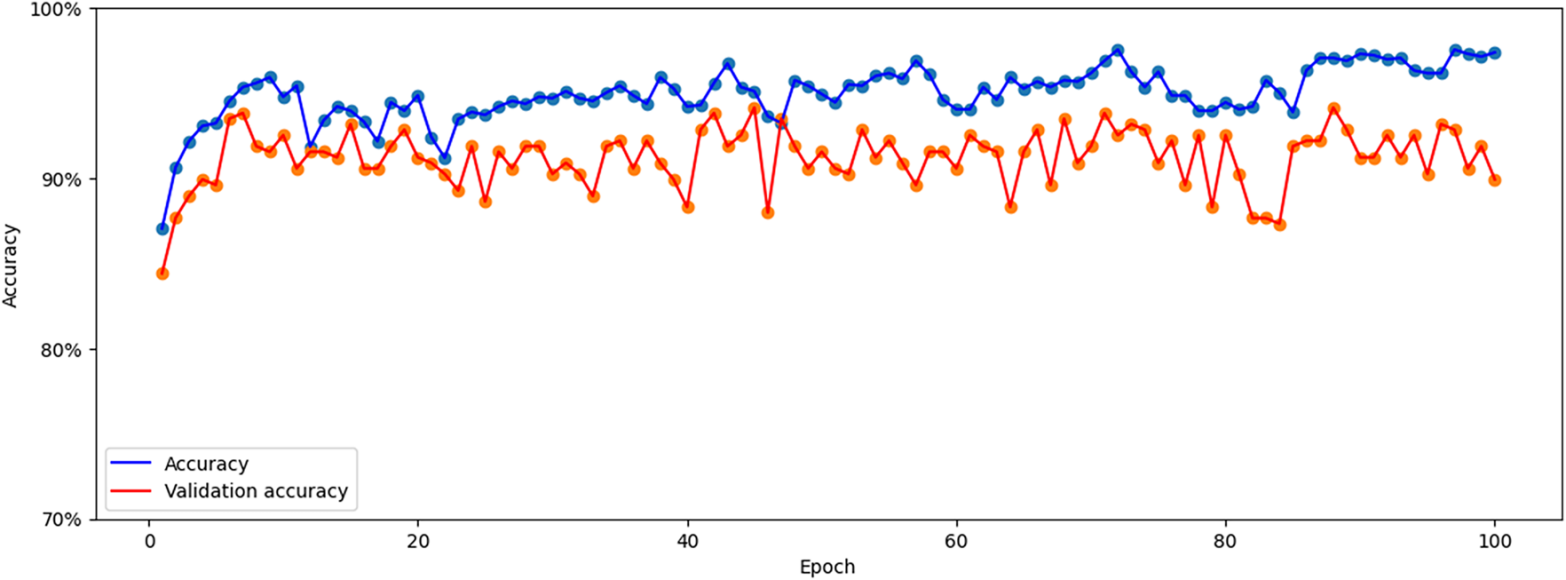
The variation of accuracy for the test and training data versus epochs.

Figures 7 and 8 represent the variations of the losses and accuracies versus epochs for the train and test data, respectively. According to these figures, it can be clearly seen that the model does not have an overfitting problem. Moreover, the accuracy and loss in training and validation data are in the same range for each epoch.

**Figure 9:**
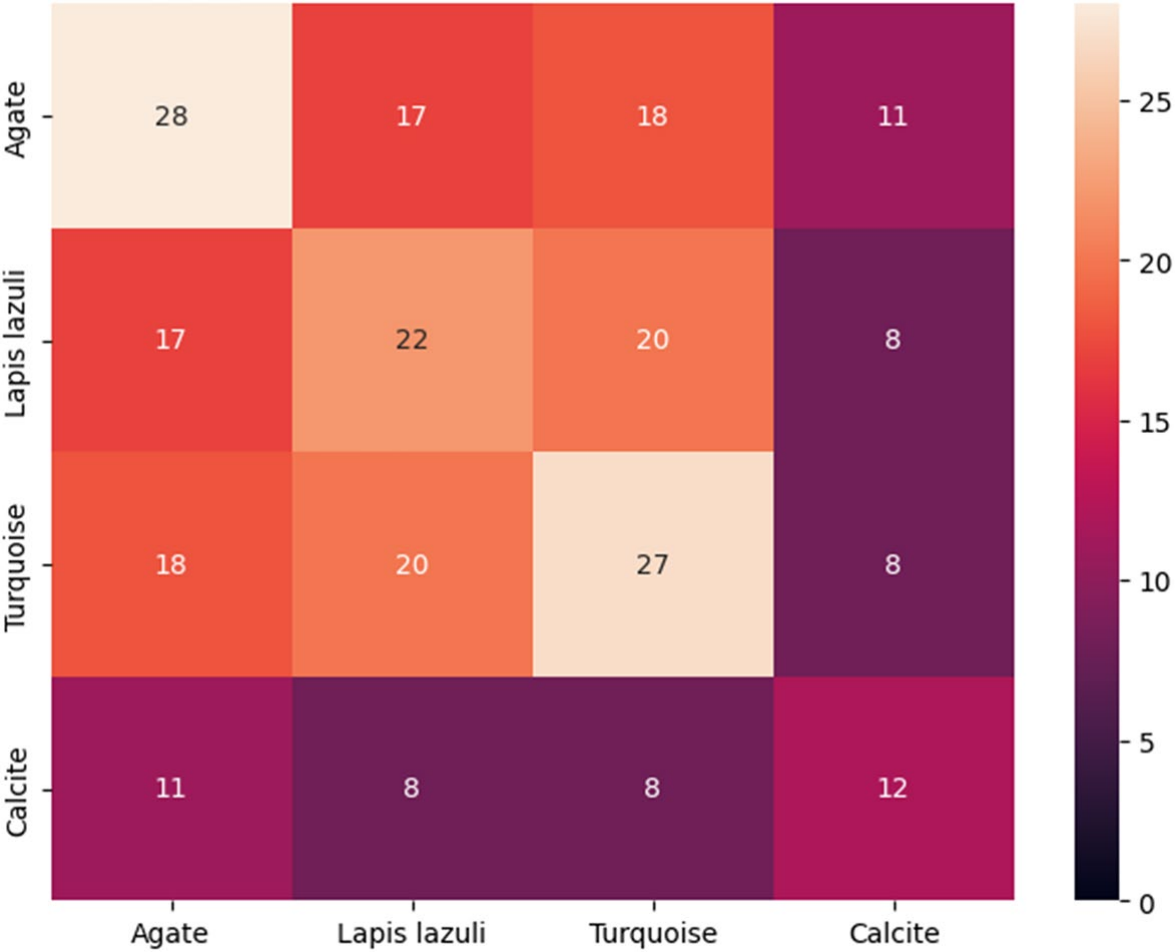
Study on common elements discovered by LIBS technique in different gemstones.

According to the obtained results, it can be concluded that the interpretability of data before modeling is a very important factor due to the transparency of the model in classification. Consequently, expensive jewelries are modeled and classified with the help of the LIBS technique and CNN–LSTM network with high accuracy. As it is seen in Fig. 9, the CNN–LSTM model for higher epochs yielded a satisfactory result, with accuracies above 98%.

The current study's findings demonstrated that jewelry stones can be discriminated based on spectral analysis using a combination of LIBS and CNN–LSTM deep learning algorithms. On the other hand, gemstones with similar appearances, but different compositions can be completely distinguished. Generally, the classified version of gemstones with exact quantitative compositions is employed in different fields, such as the jewelry industry. Therefore, classification can improve the utilization performance. The main advantage of this analysis is that no complex pre-treatment like grinding, crushing, or cutting was applied to them; just a few micrograms of gemstones were ablated. Furthermore, fast real-time detection is another point that influenced the choice of this technology.

### The interpretation of effectiveness in feature learning

To check the interpretability of results, the most important part of the experiment, i.e. the elements discovered from expensive stones with the help of LIBS technique, which is one of the features of modeling is discussed. Here, about 39 different elements are discovered with the aid of LIBS method in this experiment. It can be understood according to Fig. 9 that various gemstones have common elements. This can be one of the reasons that modeling with the help of deep learning cannot perform well in classification.

**Figure 10:**
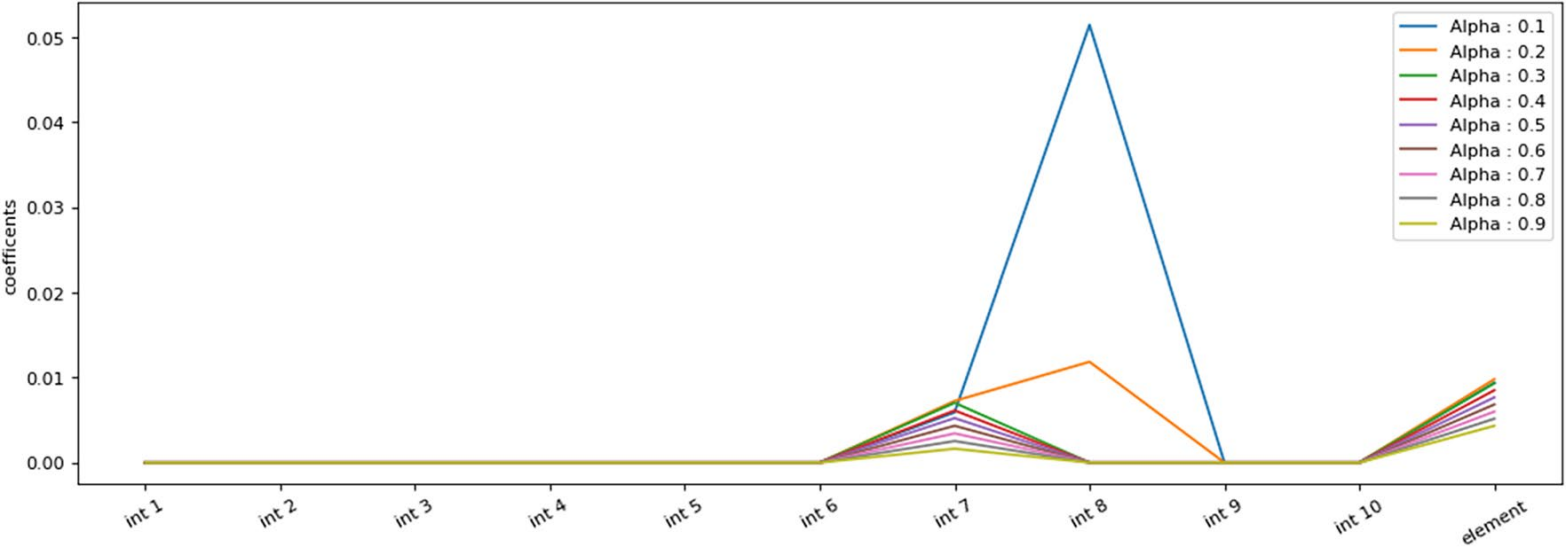
Evolution of the effective values of each feature for modeling with different values of adjustment coefficient.

**Figure 11:**
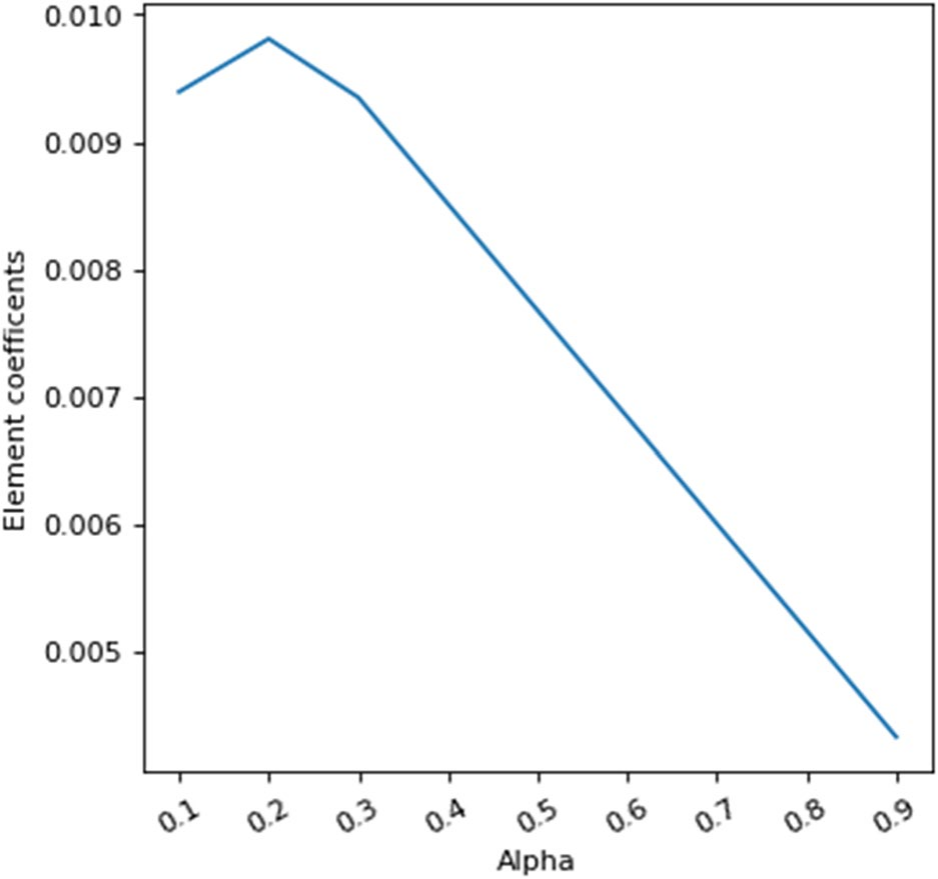
The evolution of the rate of the effective value of the element feature discovered by LIBS based on the different values of the adjustment coefficient.

Figures 10 and 11 investigate the effective value of the feature element discovered by LIBS technique with the help of LASSO method employing different values of the adjustment coefficient. As it is clearly seen in these figures, by increasing of the adjustment coefficient, the effective coefficient of the influence of the feature element is decreasing linearly. This indicates the high importance of this feature in classification.

**Figure 12:**
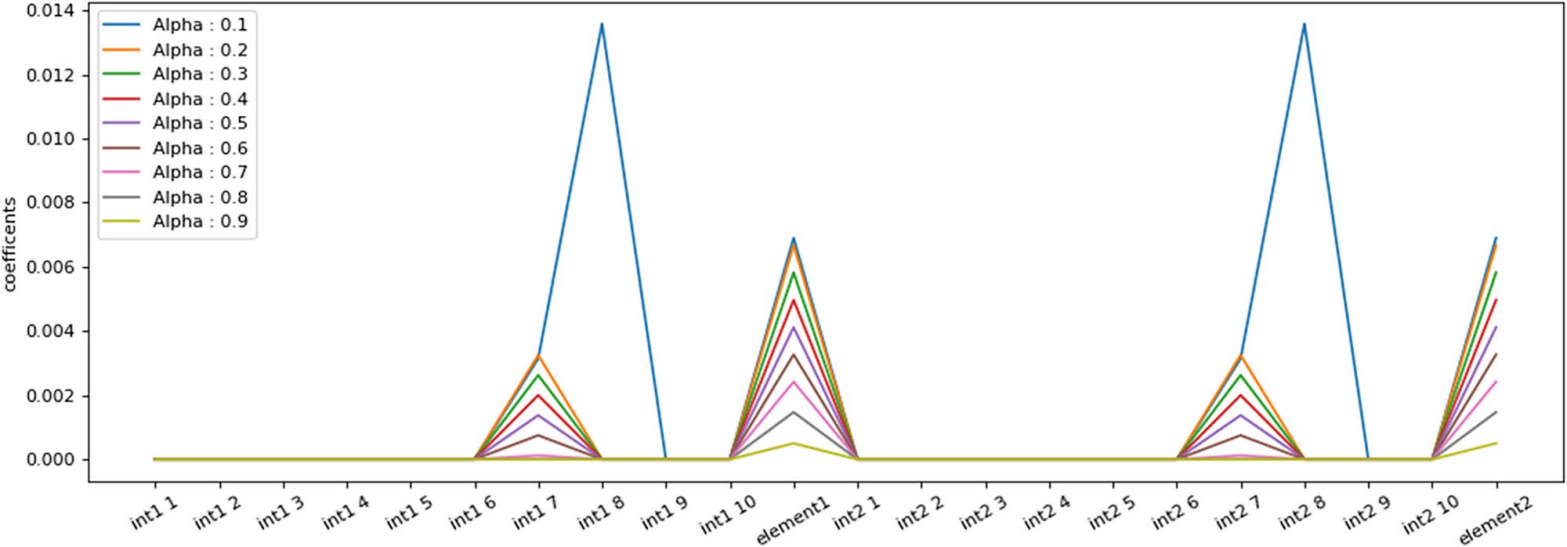
Results of the different adjustment coefficients between 0.1 and 0.9.

**Figure 13:**
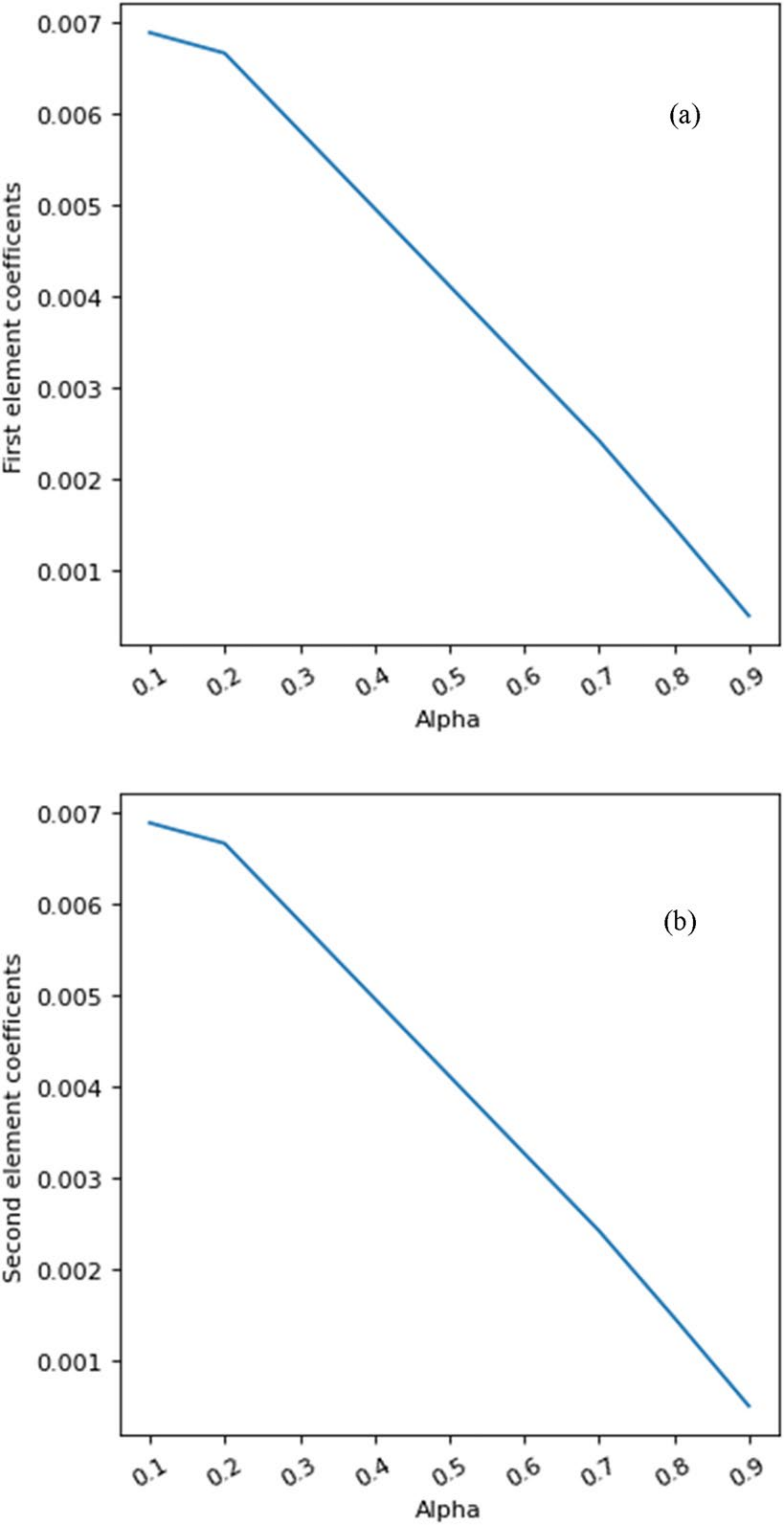
The variation of the (**a**) first, and (**b**) second element coefficient versus alpha.

In following, all the input data related to the different gemstones have been combined. This means that for classification, instead of one input spectrum and one identified element, two input spectra have been used with two elements identified using LIBS method. Then, with the help of LASSO algorithm, the effect of the features of input element 1 and input element 2 with different adjustment coefficients has been checked. Figure 12 shows the distribution of the different adjustment coefficients including line intensities and elements between 0.1 and 0.9. As it is seen in this figure, adjustment coefficients represent some peaks at similar intensities in all of alpha magnitudes, except at 0.1. In addition, by decreasing alpha values, the adjustment coefficients enhance so that when alpha equals to 0.1, the greatest magnitude for adjustment coefficient happen. Furthermore, Fig. 13 presents the evolution of the first and second element coefficient versus alpha. As it is clearly observed in this figure, by increasing the alpha parameter, both of first and second element coefficients decrease.

Generally, the Figs. 12 and 13 check the weight value of the linear model of each feature with different value of alpha. Lasso is used to make the model regulated, so that if it is zero, it has no effect, and if it is equal to 1, the coefficients of linear weight of all features are zero. These diagrams show that the feature of the element discovered by LIBS has an effective weight factor in the model and a very important and effective feature in modeling is the ratio of intensities. At the same time, the importance of none of these elements has not decreased with the combination process.

### Validity of results and making comparisons

To demonstrate how effective this method is in data analysis, its performance is compared to the results reported in various published literatures^51–56^. In almost all of the research^51–53^, digital images are utilized to accurately detect the composition of gemstones and discriminate between those using deep Convolutional Neural Networks. For instance, Ref.^51^ employed machine learning algorithms with respect to the image processing for the classification of different jewelry stones. They compared 82 state-of-the-art machine learning techniques’ algorithms for this purpose. Their accuracies were variable, ranging from 0.4 (for protoclass and oblique tree algorithms) up to 1 (for the KNN method). Furthermore, in Ref.^52^, the efficiency of a computer-vision-based method is compared against that of trained gemmologists for the classification of various images for about 68 classes of jewelry stones. They examined 9 algorithms: Random Forest, Logistic Regression, Support Vector Machine, ResNet50, Naive Bayes, ResNet18, Linear Discriminant Analysis, K-Nearest Neighbor, and Decision Tree, and reported the accuracies between 42.6 and 66.9%. In addition, in the LIBS technique, gemstones have been mostly classified by the principal component analysis (PCA) algorithm^54–56^. Therefore, by making a comparison, it can be concluded that the Convolutional Neural Networks assisted LIBS technique can considerably improve the discrimination process with accuracies approximately higher than 90 percent and that the methodology of CNN–LSTM can be replaced by other traditional algorithms in LIBS. Additionally, the interpretable dynamical trends of data helped the accuracies of analysis.

The maximum accuracy reported for jewelry stone classification in previous literature was not as high as the present paper. Consequently, if there were several numbers of unknown samples with the same compositions, LIBS spectrum data alone may take a longer time to differentiate, but with the aid of the deep learning analysis of the LIB spectral analysis, the classification can be done rapidly. On the other hand, any kind of unknown jewelry stone can be quickly and simply identified using CNN–LSTM of the LIB spectral data if a library of known gemstone samples is available. Finally, as an excellent representative of deep learning, the Convolutional Neural Network (CNN) is a superior method in feature learning and data fitting. Recently, it has incrementally introduced itself in spectral analysis.

## Conclusion

With the potential for extension of real-time chemical analysis in the field for several geological, environmental, archaeological, and forensic applications, laser-induced breakdown spectroscopy (LIBS) has been proposed as a chemical sensor technology. In this study, we show that LIBS can be a useful tool for gemstone identification and discrimination through a “gemstone fingerprinting” approach. Here, different jewelry stones including agates, turquoises, calcites, and azures related to Shahr-e Sukhteh (the Burnt City) in Iran, are classified by a combined LIBS and convolutional LSTM algorithm. Lasso method was applied on spectral data as a factor for investigation of interoperability.

Numerous experiments were performed to confirm the effectiveness of the suggested model. We have shown that, compared to other common discrimination methods, the constructed convolutional LSTM method outperforms other techniques.

In addition, the results demonstrated that CNN–LSTM accuracy was very high for different gemstones of agate, turquoise, calcite, and azure. The findings also showed that the machine learning assisted LIBS technique can play a crucial role in ensuring rapid, precise, and excellent classification. We have shown that LIBS technology combined with machine learning, can quickly and accurately classify jewelry rocks which may be further developed to applied in the jewelry industry.

## Data Availability

The datasets used and analyzed during the current study available from the corresponding author on reasonable request.
